# Emerging electrolyte-gated transistors for neuromorphic perception

**DOI:** 10.1080/14686996.2022.2162325

**Published:** 2023-01-11

**Authors:** Cui Sun, Xuerong Liu, Qian Jiang, Xiaoyu Ye, Xiaojian Zhu, Run-Wei Li

**Affiliations:** a CAS Key Laboratory of Magnetic Materials and Devices, and Zhejiang Province Key Laboratory of Magnetic Materials and Application Technology, Ningbo Institute of Materials Technology and Engineering, Chinese Academy of Sciences, Ningbo, China; b Zhejiang Province Key Laboratory of Magnetic Materials and Application Technology, Ningbo Institute of Materials Technology and Engineering, Chinese Academy of Sciences, Ningbo, China; c College of Materials Sciences and Opto-Electronic Technology, University of Chinese Academy of Sciences, Beijing, China

**Keywords:** Electrolyte-gated transistors, artificial synapse, artificial neuron, neuromorphic devices, int elligent perception

## Abstract

With the rapid development of intelligent robotics, the Internet of Things, and smart sensor technologies, great enthusiasm has been devoted to developing next-generation intelligent systems for the emulation of advanced perception functions of humans. Neuromorphic devices, capable of emulating the learning, memory, analysis, and recognition functions of biological neural systems, offer solutions to intelligently process sensory information. As one of the most important neuromorphic devices, Electrolyte-gated transistors (EGTs) have shown great promise in implementing various vital neural functions and good compatibility with sensors. This review introduces the materials, operating principle, and performances of EGTs, followed by discussing the recent progress of EGTs for synapse and neuron emulation. Integrating EGTs with sensors that faithfully emulate diverse perception functions of humans such as tactile and visual perception is discussed. The challenges of EGTs for further development are given.

## GRAPHICAL ABSTRACT

**Figure:**
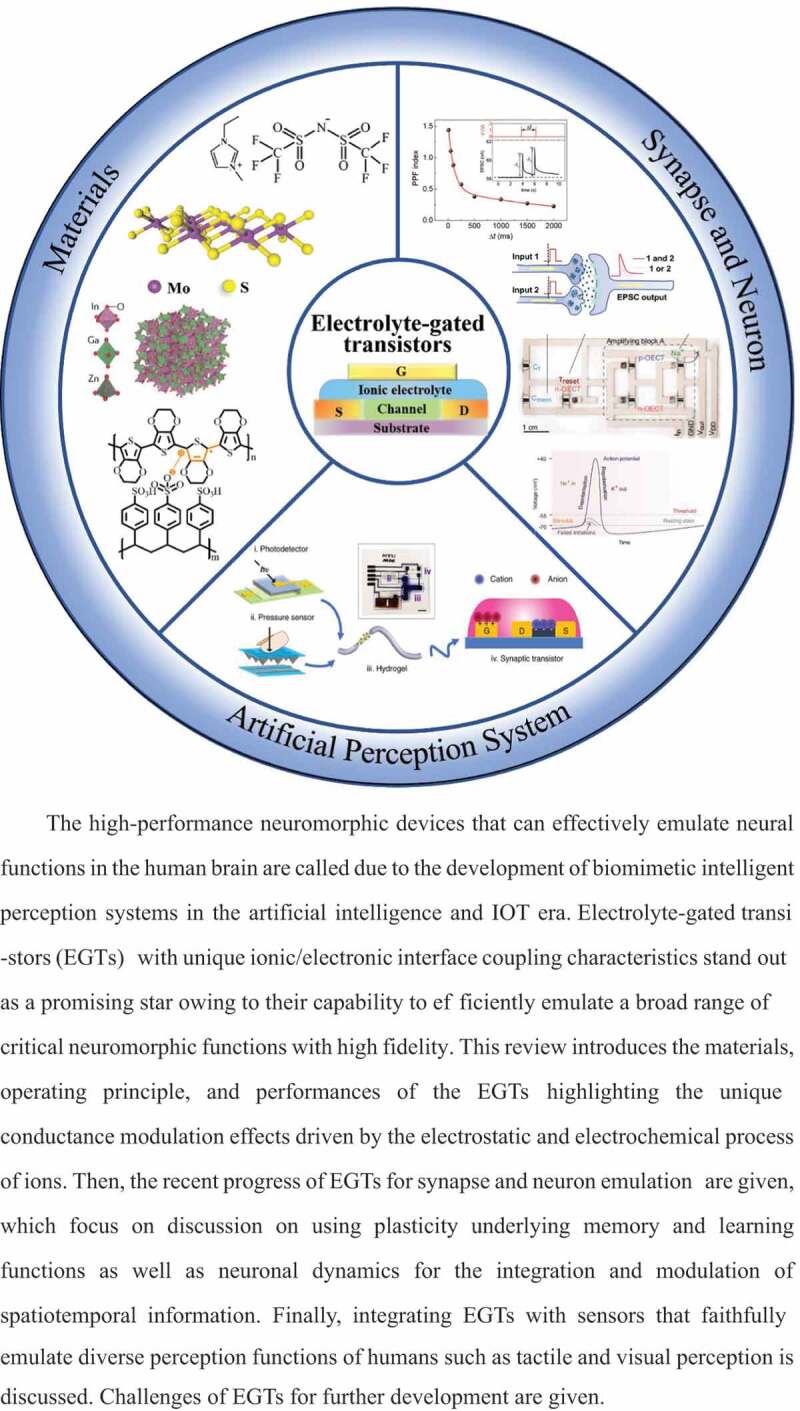

## 1. Introduction

Intelligent perception is one of the key functions of the human body, which enables the interaction of human with the external environment and understanding of the world [1]. Successful emulation of intelligent perception functions with the electronic system would significantly advance the development of robotics, neural prosthetics, and smart wearable technologies, etc. [2,3]. In biology, the perception system consists of the peripheral nervous system (PNS) and the central nervous system (CNS), where sensory organs such as skin, eyes, and nose are responsible for information acquisition, and the neural system plays the role of information processing, implementing learning, memory, and identification tasks [4–6]. To date, various high-performance sensors have been developed to mimic human sensory functions [7–12]. For instance, a flexible tactile sensor could sensitively discriminate and track the movement of ants [10], and advanced visual sensors are able to detect infrared and ultraviolet light [11], beyond the sensing limitations of human skin and eyes, respectively. In contrast, biomimetic intelligent processors that can faithfully execute signal conversion, storage, and transmission, etc., that mimic biological neural systems, have been far from meeting the requirements.

The key to developing a neuromorphic system is to mimic neurons and synapses that are the basic components and functional units of neural systems [6]. Neurons receive spatiotemporal stimuli from multiple neurons and transmit the stimuli to and integrate in the cell body, which could generate an action potential as the output in suitable conditions. Synapses are the functional connection between neurons, in which the information can be stored and processed by adaptively adjusting the connection strength of synapses. The neural system is event-driven and processes information with in-memory computing architectures and in a massively parallel manner, enabling low power consumption, high fault tolerance, and efficient information processing [13].

Digital computing units based on complementary metal oxide semiconductor (CMOS) have been proposed to simulate the functions of biological synapses and neurons [14]. However, such system deals with information essentially through physically separated computing and memory units in serial sequences, and the computing paradigm results in significant energy consumption, speed constraints, and high hardware overhead. This challenge becomes more serious with the explosive growth of data amount for processing, detrimentally affecting the efficiency of the perception system. New and disruptive computing device technologies that meet the demands of the information electronics and various applications are desired. Electrolyte gated transistors (EGTs) have been identified as one of the most promising neuromorphic devices for neuron and synapse emulation [15–19]. They exhibit unique ionic/electronic interface coupling characteristics using ionic conductor electrolyte as gate dielectric. The rich physiochemical effects including electrostatic/electrochemical regulation generate diverse conductance modulation behaviors that offer opportunities to emulate neural functions. EGTs also possess advantages including low power consumption, easy operation, and good reliability, etc. The resemblance of the underlying mechanism regarding ion redistribution with the biological counterpart further inspired the development of bio-plausible processors that inherit biological details.

In this review, we briefly discuss the research progress of using EGTs-based neuromorphic devices for intelligent perception systems. Specifically, we discuss the working mechanism of EGTs, followed by the introduction of EGTs for synaptic and neuronal function emulation. The integration of EGTs with artificial neural system with sensors for intelligent bionic perception system construction is summarized. Finally, challenges and perspectives are discussed.

## 2. Electrolyte-gated transistors (EGTs)

**Figure 1.**
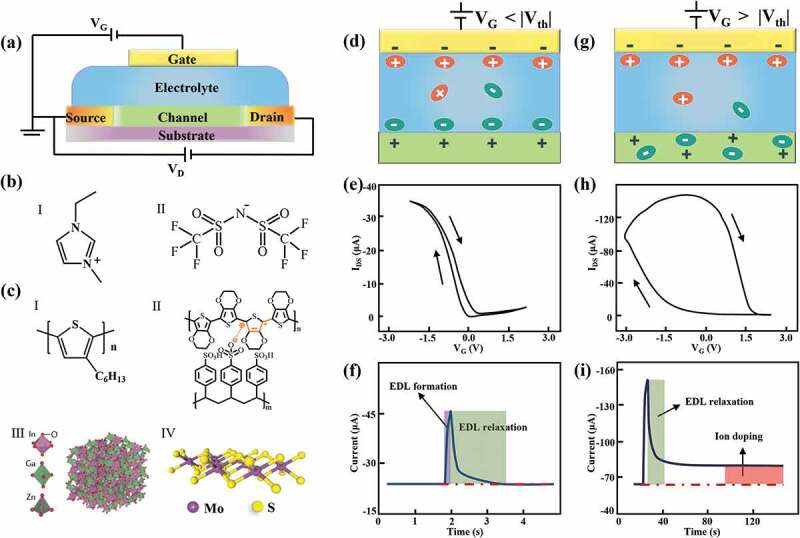
Electrolyte-gated transistor (EGTs). (a) Schematic diagram of the EGT. (b) Typical ionic electrolyte material, e.g. ionic liquid, consisted of (I) EMI cation and (II) TFSI anion. (c) Typical channel material, e.g. P3HT (I), PEDOT:PSS (II), amorphous indium-gallium-zinc oxide (III) [20] and 2D molybdenum disulfide (Ⅳ). (d–i) Schematic diagrams showing the gate voltage driven ion redistribution, the transfer curve and current response to an electric pulse, obtained from the EGTs working in the electrostatic mode (d,f,h) and electrochemical mode (e,g,i), respectively.

EGTs are similar to the metal-oxide-semiconductor field-effect transistors (MOSFET) in the configuration (Figure 1(a)) [21]. The dielectric materials use the ionic electrolyte material with good ionic conductivity and poor electronic conductivity, such as ionic liquid (IL), ion gel, polymer electrolyte, solid electrolyte [15,22,23]. IL is composed of cations, e.g. 1-ethyl-3-methylimidazolium (EMI) cation (**I**, Figure 1(b)) and anions, e.g. *N, N*-bis(trifluoromethane)sulphonamide (TFSI) anion (**II**, Figure 1(b)) [24,25]. Owing to the liquid nature of the dielectric that are challenging for manufacturing, ion-gels formed by incorporating IL into polymer, polymer electrolytes consist of inorganic salt and polymer matrix [25–28], as well as solid-state electrolytes having good Li+ and Na+ conductivity, are more widely used [29–32]. The channel materials of EGTs typically adopt dielectrics that can accommodate ions, such as organic materials (e.g. P3HT and PEDOT:PSS), amorphous metal oxides (e.g. indium – gallium–zinc oxide), 2D materials (e.g. two-dimensional molybdenum disulfide) and carbonaceous materials (I-Ⅳ, Figure 1(c)) [20,33,34–37]. During device operation, gate voltage drives the migration of ions in the electrolyte with an electric field, leading to cation/anion accumulation at the electrolyte/channel interface [15–17,37]. Depending on the impact of the applied voltage that affects the status of ions at the interface, distinct ionic processes, *i.e*. electrostatic and electrochemical carrier doping/de-doping, take places. Figure 1(d) shows the schematic diagram of the EGTs in the electrostatic mode [16,38]. Under a small V_G_, charged ions accumulated at the electrolyte-channel interface induce the opposite polarity carriers in the channel material, resulting in the formation of electric double layer (EDL). The EDL has a large capacitance value in the range of 1 to 500 *μ*F cm^−2^ due to the small interlayer distance (~nm), allowing efficient modulation of carrier concentration thus conductance of the channel layer. After the gate bias is removed, ions at the interface diffuse back to the ionic electrolyte shortly with ion concentration gradient, restoring the channel conductance to the pristine state. This process is volatile, thus producing I_DS_-V_G_ curve with negligible hysteresis (Figure 1(e)) [15,38,39]. The device response to a gate voltage pulse is shown in Figure 1(f), in which the pulse produces a transient current during stimulation, which rapidly decay to the initial state within a few milliseconds after pulse removal [39,40]. On the other hand, in the electrochemical mode with a large V_G_ applied, the ions accumulated at the interface can penetrate into the channel material (Figure 1(g)), thereby tuning the electronic states and conductivity of the channel material *via* ionic doping. Taking WO_3_ channel material [41] as an example, protons in the ionic liquid are driven towards the channel layer and penetrate into WO_3_ through an electrochemical reaction, forming H_x_WO_3_. The doping of WO_3_ induced by protons increases the conductivity of the channel and leads to significant conductance changes. Owing to the fact that the injected ions can be stably preserved by the channel dielectric due to the existence of energy barrier for ion migration and redox reaction, the conductance modulation is nonvolatile and a large hysteresis during I_ds_-V_g_ sweeping is generally observed [37,42–44] between the forward and reverse traces in the I_DS_-V_G_ curve (Figure 1(h)) [15,38]. A reverse voltage is needed to extract the trapped ions that recover the conductance to the pristine state. The pulse stimulation dependent device response is illustrated in Figure 1(i). Therefore, the device exhibits voltalie and nonvolatile conductance modulation, which can be engineered by controlling the voltage amplitude to induce electrostatic and electrochemical process respectively [40]. Under voltage pulse stimulation with controlled polarities and strength, the device conductance can be progressively enhanced/depressed, giving rise to multiple accessible discrete conductance states [17,18]. For instance, Deng et al. [40] report an EGT using a rubbery solid ionic gel and VO_2_ as the electrolyte and channel material, where the reversible Li-ion intercalation and de-intercalation in VO_2_ film tune the device conductance. By applying positive and negative gate pulses (+1.8/-1.2 V, 2 s), 120 discrete conductance states with conductance ranging from 75 to 350 nA can be obtained. van de Burgt et al. [45] developed a PEDOT:PSS-based electrochemical organic device (ENODE) exhibiting 500 discrete, highly reproducible and long-term stable conductance states through (+10/−10 mV, 1 ms) potentiation and depotentiation pulses gating. This excellent performance originates from the reversible and stable protonation/deprotonation of PEI, which tunes the concentration of holes in the PEDOT skeleton under gating voltage [46,47]. Generally, the energy consumption of EGTs is orders of magnitude lower than that of CMOS transistors. For instance, Li et al. [48] demonstrate that in EGTs employing insulating *α*-Nb_2_O_5_ as the channel layer, the power consumption is approximately 20 fJ μm^−2^. The programming energy is proportional to the channel size that can be further reduced through device minimization [17,49]. Xu et al. [50] report an organic nanowire transistor with 300-nm channel length, attaining ~1.23 fJ per spike, comparable to that of biological systems.

## 3. Neuromorphic functions emulated with EGTs

Learning, memory, forgetting, and associated cognitive activities are completed by complex neural networks that serve as the most important part of the perception system. The neural system of the brain consists of approximately 10^11^ neurons and 10^15^ synapses, which enables processing of large amounts of information, making the brain one of the most efficient computing systems in nature [51,52]. A deep understanding of the biological neural system would undoubtedly promote the development of neuromorphic systems with high fidelity [53]. This section introduces the functions of the biological neural systems, and focuses on the research progress of EGTs in the emulation of synapses and neurons, which can provide guidance for the exploration of high-performance neuromorphic devices.

### 3.1 Synaptic function emulation

**Figure 2.**
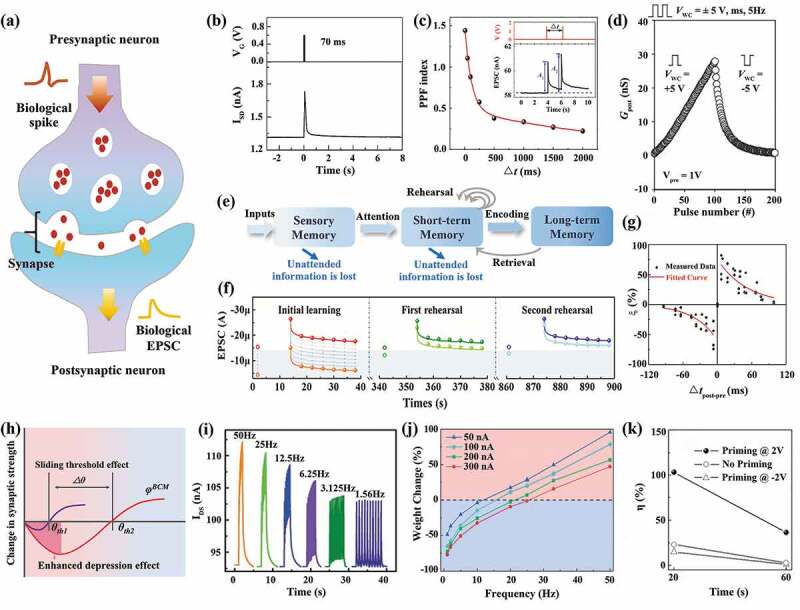
Homosynaptic plasticity emulated with EGTs. (a) Schematic illustrating a biological homosynapse. (b,c) Short-term synaptic plasticity and paired pulse facilitation (PPF) functions emulated with EGTs. Reproduced by permission from [41,54], copyright [2018, John Wiley and Sons]. (d) Long-term synaptic plasticity emulated with EGTs. (e,f) the flowchart and experimental results demonstrating STM-LTM transition. (g) STDP functions emulated using an EGT. Reproduced by permission from [55], copyright [2018, John Wiley and Sons]. (h) Schematic diagram showing the BCM effects in biological synapses. (i) the SRDP characteristics. Reproduced by permission from [40], copyright [2021, John Wiley and Sons]. (j) Sliding frequency threshold effect [56]. (k) Mimicry of the metaplasticity behavior [57].

Synapses (Figure 2(a)) are the connection part among neurons for information transmission and storage. The connection strength that determines signal transmission efficiency between pre- and post-neurons can be either enhanced or weakened through different biology ionic processes, corresponding to the synaptic plasticity. Synaptic plasticity forms the foundation of learning and memory [58,59]. In general, multiple synapses are interconnected together in biological systems, and a given synapse can possibly be influenced by the stimulus signals applied to it or its neighboring synapses. Depending on whether the input signal is specific, synaptic plasticity is divided into homosynaptic plasticity and hetero-synaptic plasticity.

#### 3.1.1 Homosynaptic plasticity

When emulating the homosynaptic plasticity, the gate terminal and source-drain terminal play the role of pre- and post-synaptic terminal, respectively, where electrolyte corresponds to the synaptic cleft. The EGT can emulate the short-term and long-term plasticity of a synapse for transient and persistent memory formation [18]. Specifically, short-term synaptic plasticity refers to volatile synaptic weight changes, where synaptic weight decays over time (ms ~ s) after enhancement/weakening [19,60]. This behavior can be emulated with EGTs in the electrostatic mode, where the ions accumulate at the dielectric/channel interface during pulse stimulation and spontaneously diffuse back after pulse removal, leading to an initial increase and subsequent relaxation of the conductance (Figure 2(b)) [41]. Paired-pulse facilitation (PPF) depicts the key characteristics of the short-term plasticity response to the paired electric pulses, where the response to the second pulse is higher than the first one. It can be well implemented by carefully engineering the pulse interval that controls the accumulation of residual ions at the interface [59,61]. Yang et al. [54] applied a pair of voltage pulses (1.5 V, 10 ms) to Li ion-based all-solid-state EGT to emulate the PPF. The ratio between the second excitatory post-synaptic current (EPSC) current and the first EPSC one is defined as the PPF index. It was found that the shorter the pulse interval is, the higher the PPF index is, thus the more prominent the enhancing effect is (Figure 2(c)). The short-term memory (STM) effects facilitate the extraction of temporal features in the dynamic inputs [58–60].

Long-term synaptic plasticity refers to the synaptic efficacy changes lasting for hours or longer, emulating the prolonged information memorization. This can be achieved by operating the EGTs in the electrochemical mode for nonvolatile modulation of the channel conductance. Oh et al. [62] report a silicon-indium-zinc-oxide/ion gel-based synaptic transistor with excellent long-term potentiation (LTP) and long-term depression (LTD) characteristics. The steady increment of the device conductance from 0.6 to 27.8 nS is achieved by applying a series of positive pulses to the gate, regarded as the LTP. After stimulating the gate with negative pulses, the gradual decrease of the conductance can be obtained, mimicking the LTD (Figure 2(d)). The long-term memory effects are recognized as a fundamental mechanism for stable learning and memory, which can be leveraged for target feature storage.

Through repeated stimulation, the memory could be strengthened and transited from STM to LTM in biology (Figure 2(e)) [63,64]. This can be realized in EGTs by increasing the pulse stimulation cycles that improve the retention performance [65,66]. Liu et al. [67] fabricated a P3HT/PEO nanowire-based neuromorphic transistor to simulate the STM-LTM transition. Nine consecutive groups of presynaptic spikes were used to regulate the triggered EPSCs of the device. After the first consecutive groups of the learning process, the fast conductance decay indicating the quick forgetting and the STM characteristics is detected (Figure 2(f)). Subsequent device stimulation with another consecutive group of pulses drives the memory level of knowledge to a higher current level with enhanced memory retention, exhibiting the LTM effect (Figure 2(f)). This transition is likely caused by the repeated accumulation of ions at the interface that promotes ion penetration into the channel material, switching of the device from the electrostatic to the electrochemical mode.

Spiking time-dependent synaptic plasticity (STDP) [68] and Bienenstock-Cooper-Munro (BCM) are two typical learning rules found in biological synapses. For the former case, the change in the synaptic connection strength is not only related to the signals from the pre-synaptic neuron but also affected by the activity of the post-synaptic neuron. It describes a spatiotemporal biological process, in which the long-term connection strength between neurons can be regulated by the relative timing of pre- and post-synaptic spikes, and plays a vital role in determining the polarity and magnitude of the weight changes [54,69–73]. Depending on the plasticity tuning characteristics, four types of STDP behaviors have been demonstrated by EGTs, including Hebbian STDP, anti-Hebbian STDP, symmetrical STDP, and visual STDP. For instance, Yu et al. [55] successfully emulated STDP using the EGT device in which chitosan-based electrolyte with a high proton conductivity was used as the dielectric layer, and indium-tin-oxide (ITO) was utilized as the channel layer. By carefully adjusting the voltage parameters of pre- and post-synaptic spikes that tune the short-term and long-term device dynamics, the STDP effect is successfully replicated (Figure 2(g)).

Another fundamental activity-dependent learning rule is BCM learning rule (Figure 2(h)) which elaborates that the frequency-dependent synaptic polarity modulation behaviors, including spike-rate-dependent plasticity (SRDP) and a sliding frequency threshold effect (*f*) [74–80]. Spike-rate-dependent plasticity (SRDP) showing the enhanced weight changes with increased pulse frequency is achievable with the EGTs. Deng et al. [40] applied 10 repetitive pulse stimulus with different pulse frequencies to an ionic gel-gated VO_2_ transistor. A stepwise enhancement of EPSC can be found at high frequency, and the EPSC enhancement gradually weakens with the decrease in the pulse frequency (Figure 2(i)). Biologically, high spiking rates exceeding a frequency threshold can strengthen synaptic weight, whereas low spiking rates below the threshold weaken synaptic weight. Thus, the sliding frequency threshold (*f*) that determines whether the response is depression or potentiation, controls synaptic strength. Huang et al. [56] develop a polysaccharide-gated synaptic transistor with *λ*-carrageenan and ITO as the channel layer, the protons drift to the electrolyte/channel interface during pulse stimulation, and diffuse back to the electrolyte after pulse removal. The net amounts of accumulated ions at the interface are determined by the competition of the ions drifting in and diffusing out. A high/low pulse frequency leads to the enhancement/weakening of the synaptic weight. As the initial channel current increases, the frequency threshold shifts towards the high-frequency direction, resembling the frequency sliding character of BCM as shown in Figure 2(j).

In biological systems, neural activity at present state is history dependent and can be recessive. If an episode of priming stimulus was applied to synapses, their ability to evoke the synaptic plasticity is altered even after a later bout of activity. This form of plasticity regulation refers to synaptic metaplasticity. Metaplasticity highlights the importance of the previous history of activities on subsequent synaptic plasticity, where the effect persists to induce subsequent synaptic plasticity and does not necessarily influence the current synaptic weight [57,81–88]. To study the effect of priming stimuli on the synaptic weight, Ren et al. [57] proposed nano-granular phosphorus silicate glass (PSG)-based proton conductive electrolyte-gated oxide neuromorphic transistor. Without priming spikes, the change in synaptic weight is ~23%, which shifts to ~15% and ~103%, respectively, with priming spikes of −2 and 2 V, respectively. The influence lasts for 60 s (Figure 2(k)). This observation may be caused by the modified ion distribution in the electrolyte induced by the priming gating stimuli that affect the subsequent ion modulation and device responses.

#### 3.1.2 Heterosynaptic plasticity

**Figure 3.**
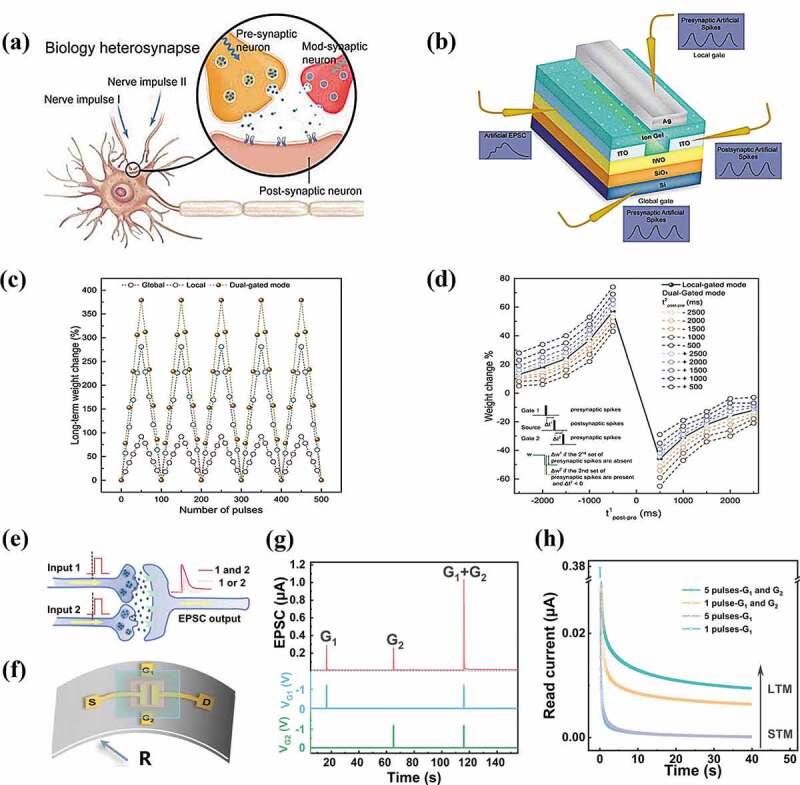
Heterosynaptic plasticity emulated with EGTs. (a) Schematic illustration of a biological heterosynapse. Reproduced by permission from [89], copyright [2019, John Wiley and Sons]. (b) The EGTs with front and back gates for heterosynapse emulation. (c,d) Experimental demonstration of adjustable (c) LTP/LTD and (d) STDP behaviors through heterosynaptic gating [90]. (e) Schematic showing a heterosynapse excited by electric inputs transmitted by two presynaptic terminals and the resulting EPSC as an output. (f) Schematic of an EGT with coplanar gates. (g) EPSCs excited by the presynaptic spikes applied on the gates. (h) Time-dependent read current of the electrolyte gated transistor after different stimuli conditions [91].

Heterosynaptic plasticity stresses that the activity of a modulatory neuron can strongly affect the connection between the neighboring neurons, crucial for complex memory functions such as associative learning (Figure 3(a)) [89,92–95]. In structure, the emulation of biological heterosynaptic functions demands multiple terminals that can concurrently modify the channel conductance. Heterosynaptic plasticity plays an enormous role between local activity and global neuromodulation observed in biological neural systems, and its mimicry offers opportunities to reproduce more complex synaptic functions [96–104].

John et al. [90] designed a dual-gated architecture (Figure 3(b)) with top and back gate terminals to emulate the heterosynaptic behaviors. Ag and ion gel are used as the top-gate electrode and dielectric, respectively, where the channel conductivity can be tuned through the voltage gated electronic-ionic coupling at the dielectric/indium-tungsten oxide (IWO) channel interface. On the other hand, Si and SiO_2_ are used as the back gate electrode and dielectric layer with carrier trapping/de-trapping effects that controls the channel conductivity. Serving as the modulatory synaptic terminal, the top gate is found to be able to modify the polarity and magnitude of the back gate controlling device conductance (Figure 3(c)). Therefore, by carefully engineering the gating conditions on the dual gates, the channel conductivity could be either enhanced or suppressed, well mimicking the modulation effects of the heterosynapses (Figure 3(d)).

The multi-terminals can also act as separate synaptic inputs that interact with each other to complete the synergistic learning and memory task. Liu et al. [91] reported an artificial heterosynapse based on coplanar dual-gate with spatiotemporal information integration and storage capability (Figure 3(e,f)). They show that the electric pulses applied on the single gate or unsynchronized electric pulses applied on the dual gates only induce volatile conductance modulation effect for short-term memory emulation. In contrast, the electrical pulse signals synchronously applied to the dual gate can lead to the cooperative formation of long-term memory to perform coincidence detection and memory function of biological heterosynapses (Figure 3(g,h)). Further simulation results show that an artificial neural system based on such hetero-synaptic devices can effectively filter the random noise signals during information learning, helping to form accurate and stable memory.

### 3.2 Neuronal function emulation

**Figure 4.**
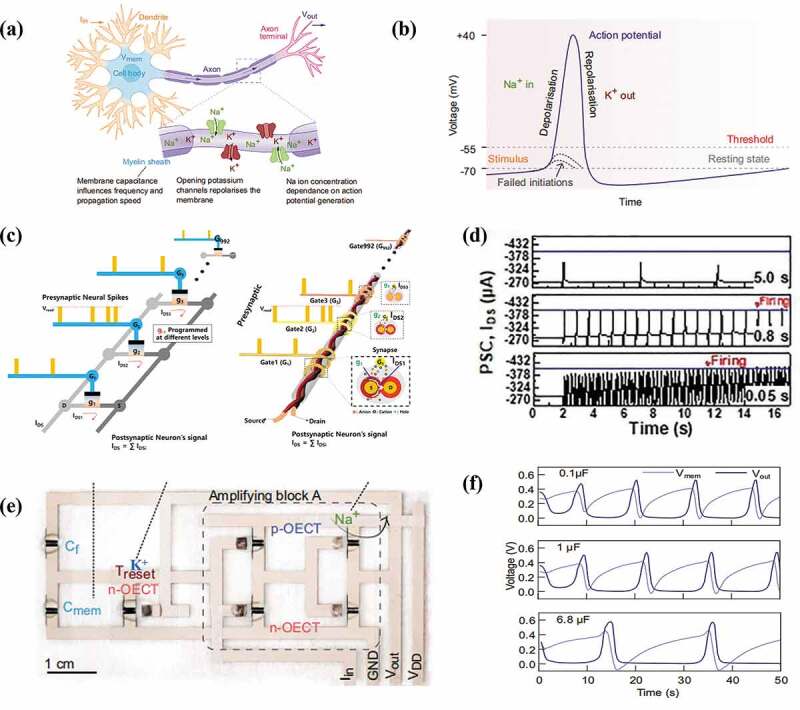
Neuronal function emulation with EGTs. (a) Illustration of a biological neuron. (b) The neuronal action potential [105]. (c) Schematic LIF neuron with EGTs. (d) The neuronal LIF behaviors emulated by the EGT, at different incoming spike frequencies. Reproduced by permission from [106], copyright [2021, John Wiley and Sons]. (e,f) An organic electrochemical transistor based artificial H-H neuron, and the produced firing patterns [105].

Typically, a neuron is composed of three compartments, namely, the dendrites, the axons, and the soma (cell body). The dendrites with a tree structure receive signals from other neurons and the axon passes the newly-generated neural spike signals as the output to other neurons. The soma functions as the key computing units for signal integration and generation (Figure 4(a)). The neurons can become inactive (at the resting state) or active (at the firing state), depending on the external stimulation conditions that control the neuronal membrane potential. For neurons at the resting state with a potential of ~-70 mV, the input neural spikes activate ion channels on the neuronal membrane that tune the flow of charged ions (such as Na+, K+, Cl−, etc.) across the cell membrane (Figure 4(b)) thus pulling up the membrane potential. When a threshold membrane potential (e.g. −55 mV) is reached, the neuron is set to the firing state and creates an action potential as the output signal [105,107,108].

Mathematically, the activation of neurons can be described by well-developed neuronal models [109], including the integrate-and-fire model (IF) [110,111], the leaky-integrate-and-fire (LIF) [106–108,112,113] model and the Hodgkin-Huxley (H-H) model [114,115], etc. The IF model highlights the monotonous change of the neuronal membrane potential during incoming spikes integration, which fires a neural spike once the threshold is reached. In contrast, the LIF model further considers the spontaneous membrane potential relaxation owing to the ion leakage during stimulation intervals, which is more biorealistic. To physically implement the LIF model, Kim et al. [106] proposed neuromorphic fibrous organic electrochemical transistors with a double-stranded assembly of electrode microfibers and multiple Au microfiber gates (Figure 4(c)). Each local area channel conductance can be modulated through one gate by ion doping/de-doping and correspondingly generate the response current which is integrated in a leaky way at the postsynaptic neuron in response to the incoming spikes. Figure 4(d) shows the LIF behavior where the device current rises with pulse stimulation and decays during pulse interval, reproducing the spike integration and leaky behaviors. When the output current reaches a predefined current level that is deemed as the threshold (blue line), an action potential was considered to be fired. The H-H model aims to capture the detailed dynamics of ion channels, *i.e*. Na+ and K+ ion channels, and are useful in producing complex neural firing dynamics. To date, few studies have explored the utilization of the EGTs for H-H model study, owing to their complexity in circuit design. Organic electrochemical neurons (OECNs) based on the EGTs and auxiliary circuit elements (Figure 4(e)) are recently developed and reported by Harikesh et al. [105]. The OECNs contain interconnected organic electrochemical transistors (OECTs) with p-type and n-type channels for Na+ and K+ ion channel emulation. The spiking frequency can be modulated in the range of 48–85 mHz by adjusting the C_mem_ capacitance in the circuit, as shown in Figure 4(f).

**Figure 5.**
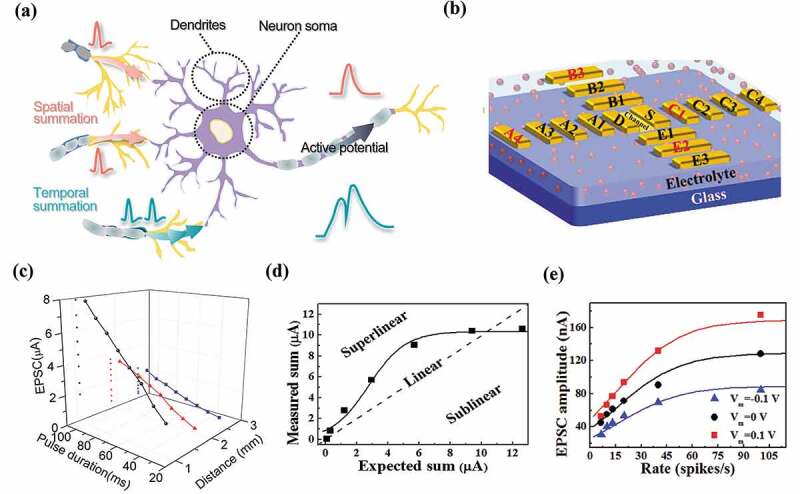
Neuronal dendritic functions emulated with multi-gate EGTs. (a) Schematic diagram of an artificial neuron consisting of dendrites, soma, and axons. Dendrites receive synaptic inputs from multiple pre-neurons. (b) EGTs with a multi-gate structure. Reproduced by permission from [116], copyright [2019, John Wiley and Sons]. (c) Spatiotemporal EPSC response in EGT. The EPSCs as a function of pulse duration and the distance between the gate and the channel (dendrite to post-synapse). Reproduced by permission from [117], copyright [2017, AIP Publishing]. (d) Different integration behaviors of synaptic inputs of the artificial dendrite. (e) The dendrite integration behaviors tuned by the gating voltage. Reproduced by permission from [118], copyright [2016, John Wiley and Sons].

While the neurons are generally modeled and simplified as a point that represents the soma for spike integration and firing, biology studies indicate that the neuronal dendrites with a branch morphology structure play an important role in spatiotemporal signal integration (Figure 5(a)) [119,120]. The EGTs with coplanar multi-gate terminal structure (Figure 5(b)) are well suited for dendritic function emulation, where the gate terminals serve as the input terminals of the dendrites whose spatial distribution can be manually modified [116–118,121]. The dendrite sums up incoming synaptic signals occurred at different spatial positions through temporal integration [116,119]. The resultant effect of two or more excitatory synaptic inputs is related to the durations and positions of these synaptic inputs on the dendrites [119]. Qian et al. [117] demonstrated a multi-gate ion-gel transistor-based artificial dendrite, and show that both the pulse duration and the distance between the in-plane gate and the channel (dendritic to postsynaptic) significantly affects the EPSC changes. Specifically, Figure 5(c) depicts EPSCs as a function of the pulse duration of 30–100 ms and dendritic to the postsynaptic distance of 1.08 to 2.64 mm. With the increase of the pulse duration, the average peak EPSC increases from 1.79 to 7.89 μA. On the other hand, when the distance increases from 1.08 mm to 2.64 mm, the slope of the spatiotemporal synaptic integration curve decreases from 0.087 to 0.028 μA/ms. This is because the increased gate-channel distance and reduced stimulation duration lead to reduced amounts of ions accumulated at the ion-gel/P3HT interface. The realization of dendritic integration at the hardware level has important implications for the further realization of neuronal functions, e.g. sound location [116,122]. According to the mathematical relationship between the current response and the arithmetic sum of the individual input responses, the integration can become linear, sublinear, and supralinear, which refers to the fact that the combined response excited by two or more inputs is identical, lower and higher than the sum of the individual response, respectively. Wan et al. [118] proposed a proton conduction-based graphene oxide neuron transistors to mimic biomimetic nonlinear integration of dendrites, where the sum of the individual responses to each input is defined as the arithmetic sum (Arithmetic Sum, S_A_) and the actually measured overall response is defined as (Measured Sum, S_M_). As shown in Figure 5(d), at low pulse voltages, the integration is almost linear. The measured sum of the intermediate pulse voltage becomes greater than the arithmetic sum (S_M_ > S_A_), triggering supralinear integration. When the pulse stimulation amplitude further increases, the sublinear integration is triggered (S_M_ < S_A_). This observation is attributed to the dynamic change of the electron mobility in the channel layer with the increased gate voltage. The nonlinear integration behaviors allow neurons to distinguish the spatial position of the received spike inputs from different neurons and has been suggested as a key mechanism for neuronal encoding/decoding [118]. The dendritic integration behaviors are also tunable by applying a modulation voltage (V_M_) on the gate at corresponding positions, which can be fitted by a nonlinear equation containing parameters governed by the input signal and modulation parameter, etc. Such modulation effect is generally known as neuronal gain (Figure 5(e)) [118,123].

## 4. EGT-based artificial perception system

During the last few years, the desire for intelligent perception systems has stimulated the growing interest in integrating neuromorphic devices with sensors for the construction of biomimetic perception systems. It is well known that biological systems have diverse receptors for different kinds of stimuli, such as pressure, light, and sound, etc. [124–126]. Receptors receive stimuli from the external environment and convert the signals into spikes, which then transmit them to the cerebral cortex for further processing. According to the types of stimuli signals that are sensed and processed, the function of the sensory system can be categorized into the mono-modal and the multi-modal neural perception.

### 4.1 Mono-modal artificial perception system

**Figure 6.**
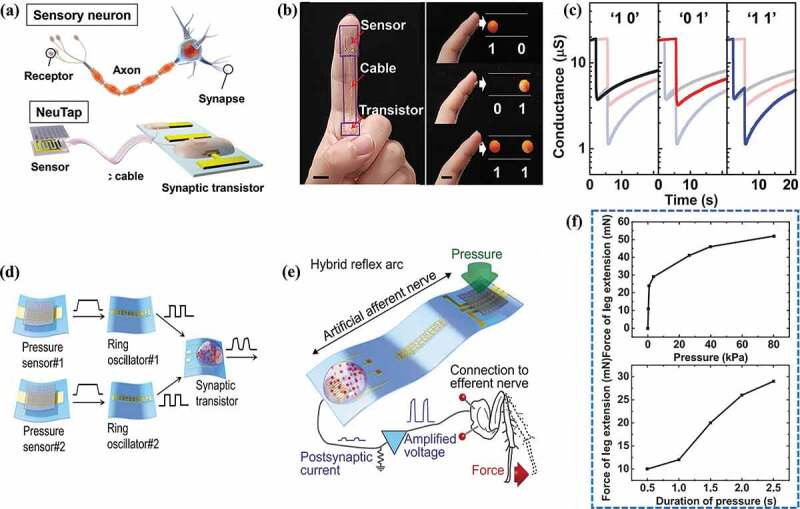
EGT-based artificial tactile perception system. (a) a sensory neuron (top) and a NeuTap (bottom). (b) the NeuTap on a finger (left) and the schematic illustrating the touch with different two-bit binary code labels (right). (c) Typical responses of the NeuTap to the touched patterns. Reproduced by permission from [129], copyright [2018, John Wiley and Sons]. (d) Artificial afferent nerve including pressure sensors, ring oscillators and synaptic transistors. (e) the hybrid reflex arc made of the artificial afferent nerve and a biological efferent nerve. (f) the isometric contraction force of the tibial extensor muscle with the intensity and duration of the stimulus application. Reproduced by permission from [130], copyright [2018, The American Association for the Advancement of Science].

Recent years have witnessed booming enthusiasm for building intelligent tactile perception system for electronic skin and intelligent prosthetics applications [127,128], inspiring the exploration of combining pressure sensors with neuromorphic devices. Wan et al. [129] reported a neuromorphic tactile sensory device (NeuTap) that mimics the tactile sensory neurons for perceptual learning. As shown in Figure 6(a), the system consists of a resistive pressure sensor, a flexible ionic cable, and a EGT-based artificial synapse, which are in analogous to the biological sensory receptors, axons, and synapses. The pressure sensor converts the pressure stimulus into electrical signals, which are transmitted to the synaptic transistor through the ion/electron interface coupling. The synaptic transistor can discriminate the difference in the temporal features of the stimulation pattern using the inherent short-term dynamics. Figure 6(b,c) shows the dynamic responses of the device to different spatiotemporally correlated haptic patterns for identification. After training, the error rate of NeuTap in pattern recognition could be reduced from ~44% to ~0.4%. Notably, the sensors in NeuTap are directly wired with the EGT, where the pressure signals are fed to the synaptic transistor without executing spiking coding. Kim et al. [130] designed an artificial afferent nerve with spike frequency coding ability by incorporating a ring oscillator, where external tactile stimuli are collected and converted into action potentials by pressure sensors and ring oscillators (Figure 6(d)). The magnitude and duration of stimulus from pressure sensors are well demonstrated by the different peaks of EPSC, *i.e*. from 2.5 to 83 kPa and from 2 to 6 sec, respectively. A detached cockroach leg serving as the actuator was further connected with the synaptic transistors to form a hybrid reflex arc, aiming to showcase the response of the intelligent perception system to the tactile stimuli (Figure 6(e)). It was shown that increasing the intensity and duration of the tactile stimulus accordingly enhances the isometric contraction force of the cockroach leg (Figure 6(f)).

**Figure 7.**
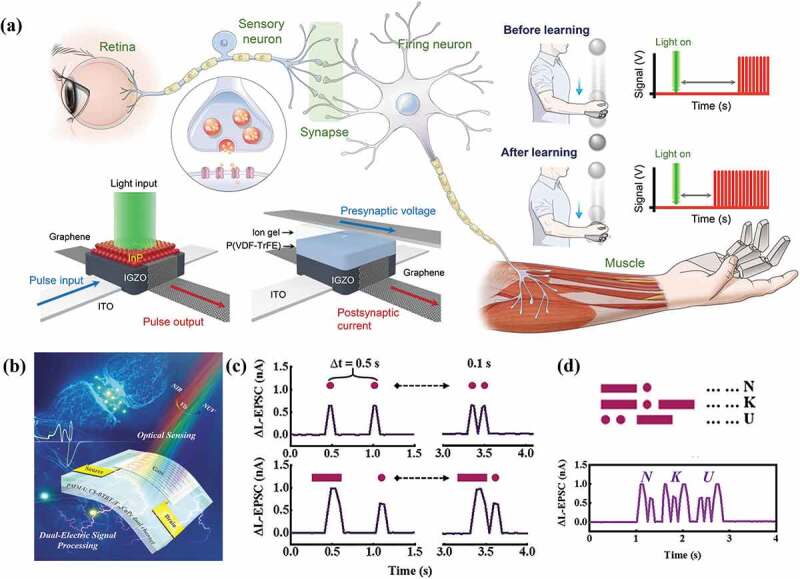
EGT-based artificial visual perception systems. (a) Schematic illustration of the biological and artificial stimulus-response system. Retina, neurons, synapses, and muscle in biological system correspond to visual receptors with InP QD layer, as with retentive EDL, an circuits and robot hand [134]. (b) Schematic of an afferent nervous system integrating broadband optical sensing and multiplexed electrical information processing. (c) the EPSC triggered by two pairs of spatiotemporally correlated 790 nm NIR light inputs over time. (d) Optical wireless communication. International Morse code of ‘NKU’ are represented by 790 nm NIR light signals of the OHNT [133].

The human visual system receives over 80% of the information from the environment. Emulation of biological visual function is important for the development of machine vision technologies that finds broad applications in autonomous driving, video analysis, and intelligent manufacturing [67,131,132]. Kim et al. [133] reported an artificial nervous system (Figure 7(a)) consisting of a quantum dot (QD) photodiode, a retentive electric double layer (EDL) transistor, and CMOS-based neuron circuits, corresponding to the visual receptor, synapse, and neuron, respectively. The visual receptor and sensory neuron circuits convert the incident light signals into electrical signals, which were analyzed by the neuronal circuit. The EGT-based artificial synapse shows the strengthened synaptic strength corresponding to LTM, after sufficient optical stimulation, which accelerates the action potential generation speed of the downstream artificial neuron. By connecting the artificial nervous system with a robot hand, an artificial stimulus-response system capable of mimicking the conscious response behavior is constructed. After sufficient training, the time required for activation of the robot hand to the light stimulus is reduced from 2.56 s to 0.23 s. This artificial stimulus-response system provides a new perspective for the development of artificial intelligence-based systems for neurological disorder patients.

Ni et al. [134] reported an ionic organic-heterojunction neuromorphic transistor (OHNT) integrated with a sensor that can detect UV, visible, and near-infrared light signals (Figure 7(b)). The integrated device exhibits selective charge-carried transport under visual or near-infrared light (NIR) inputs, which emulates the short-term and long-term memory effects of synapses. Figure 7(c) shows the EPSC triggered by two pairs of spatiotemporally correlated 790 nm NIR light inputs with different features. By encoding the International Morse code with distinct L-EPSC signals, the optical information of the three letters ‘NKU’ can be analyzed and recognized, with an accuracy of 96% (Figure 7(d)). Besides the widely reported artificial tactile and visual perceptual systems, auditory perceptual systems based on EGTs have also been explored recently. For example, Seo et al. [125] developed an artificial auditory sensory system by combining a triboelectric acoustic sensor with an ion-gated organic synaptic transistor (IGOST). The system responds to the sound wave with a frequency of 5 Hz by showing an EPSC enhancement behavior, and the device current decays rapidly (<1 s) after the sound is turned off owing to the inherent short-term memory effect.

### 4.2 Multi-modal artificial perception system

In biological systems, multisensory stimuli cooperate to enhance the ability for complex information recognition, beneficial for identification or decision-making tasks [128,135,136]. The biological multisensory system integrates five major senses of touch, sight, hearing, smell, and taste, and their interaction through neural systems in the brain allows the human to explore, learn, and adapt to the complex environment [128,135–139]. A significant advantage of biological perception systems is that they can integrate and analyze multiple types of sensory inputs, facilitating faster and more accurate analysis of information [128,135,137]. Several neuromorphic perception systems that leverage multiple types of sensors and synapses/neurons have been demonstrated.

**Figure 8.**
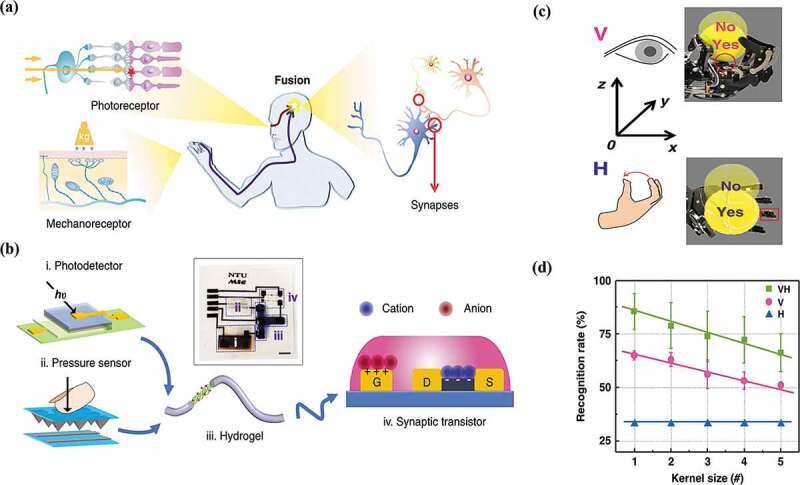
EGT-based multi-modal artificial perception system. (a) the visual-haptic fusion by biological neural system. (b) the BASE patch for visual-haptic fusion. (c) the ‘YES’ and ‘NO’ positions inferred by visual (top, pink) or haptic (bottom, blue) feedback. If the ball could be held by the robotic hand based on one sensory feedback, then the position is annotated as ‘YES’ otherwise ‘NO’. (d) the recognition rates for the mapping of mono-modal and bimodal [141].

Yu et al. [140] fabricated phototransistors with MoS_2_ and integrated flexible triboelectric nanogenerator (TENG) to provide an equivalent gate voltage to drive the synaptic transistors. It was demonstrated that synaptic transistors with multi-modal sensory functions can greatly improve the accuracy of handwriting recognition through the integration of visual and tactile information. As shown in Figure 8, Chen et al. [141] developed a bimodal artificial sensory neuron (BASE) that can fuse cues from two sensory modalities and use them for manipulation, recognition, and synaptic simulation. Photodetector and pressure sensor act as receptors in the retina and skin, which are responsible for converting external multidimensional spatial information (tactile and visual stimuli) into electrical signals. This combined current carries bimodal information in a time-dependent and nonlinear manner, very similar to neuronal behavior (Figure 8(a,b)). They also fabricated a biohybrid neuromuscular junction to transmit signals from BASE and innervate skeletal myotubes, which in turn mimicked visual-tactile fusion-based body motor control. Compared with the single sense, the fusion sense can provide high-dimensional information for the manipulator to make more accurate actions (Figure 8(c)). The bimodal sensory data can be used to identify multi-transparency letter patterns and show better performance than mono-modal sensory data (Figure 8(d)). Mimicking sensory fusion at the neuronal level has important implications for building a highly integrated perception system to improve the intelligence of robotics. Tan et al. [142] proposed an artificial multi-sensor neural system that combines five artificial senses (vision, afferent, hearing, smell, and taste) with multi-modal perception, spike encoding and memory processing capabilities, enabling the cross-modal recognition such as imagining pictures that were never-before-seen when hearing their descriptions.

## 5. Summary and outlook

The need for biomimetic intelligent perception systems in the artificial intelligence and IOT era call for high-performance neuromorphic devices that can effectively emulate neural functions in the human brain. Among diverse emerging candidates, EGTs stand out as a rising star owing to their capability to efficiently emulate a broad range of critical neuromorphic functions with high fidelity, low computing resources, and ease of integration with sensors. This article starts with the introduction of the EGT, highlighting the unique conductance modulation effects driven by the electrostatic and electrochemical processes of ions. Discussion on using EGTs with rich conductance modulation dynamics for the emulation of synaptic plasticity underlying memory and learning functions as well as neuronal dynamics for the integration and modulation of spatiotemporal information are given. Finally, the integration of EGTs with sensors and the construction of intelligent perception devices for sensory information processing is elaborated, demonstrating the great potential of neuromorphic devices in neural perception emulation.

Despite the encouraging progress, EGT-based neuromorphic computing devices are in the infancy, and there are many challenges to be solved. Firstly, the EGTs are primarily developed based on organic materials, which are unstable in ambient and moist environments, thus leading to device damage. Besides, the ionic mobility of widely used electrolyte is low, and the reversibility of ion penetration into/pumping out of the channel material is poor, which detrimentally affect the operation speed and endurance. Remarkably, recent studies show that this issue can be potentially overcome by using optimized materials such as donor-acceptor conjugated polymer and molecular engineering modified polymers as the channel dielectrics, leading to EGTs with much improved stability [143,144]. Moreover, the device suffers from uncontrollable nonideal factors, such as the variation in the operating voltages and conductance states during cycling. To mitigate these problems, careful selection, and design of the electrolyte and channel materials with good stability and engineering of the ion electrolyte/channel interface for device performance optimization are expected. Given the diversity of the electrolyte materials having been used for EGTs studies, strategies for careful electrolyte selection are important. From the perspective of function emulation, neuronal and dendritic function emulation require short-term device dynamics, thus electrolytes containing non-permeable ion compositions that tend to form volatile EDLs at the interface should be selected. On the other hand, electrolytes containing ions with high activity that can readily induce electrochemical redox reactions and long-term memory effects are preferred for synaptic function emulation. In addition, solid-state electrolytes are more attractive for large-scale neuromorphic device integration, while soft electrolytes based on ion gel are ideal candidates for flexible neuromorphic perception hardware in wearable electronics [145]. Secondly, although many basic synaptic and neuronal functions have been demonstrated in the EGTs, the implementation is mostly limited at the single device level, while few studies have targeted the array level, which is a must for application demonstration. Compared to two-terminal memristors with good scalability, the EGT with a three-terminal structure needs relatively more complex peripheral controlling circuits for the operation. Using advanced integration technologies for large-scale integration of EGTs for the construction of artificial synaptic and neural networks is a long-term goal. While growing interests have been forced on designing EGT with multi-terminal, the coplanar structure offers an alternative path for the exploration of novel neuronal dendritic functions enabled by the high flexibility in emulating the complex hierarchy of neural networks. Further exploration of the diverse dendritic effects in the EGTs is expected to give rise to novel neuromorphic devices with functions that cannot be readily achieved by two-terminal neuromorphic devices. Last but not least, to bridge the EGTs with sensors and enable the transmission of high-quality signals for processing, peripheral circuits for signal amplification and de-noise and neuronal circuits for spike coding are needed. This architecture comprises digital units, and the redundant data movement, conversion, and pre-processing cannot be circumvented. Further optimization of the integration schemes, e.g. replacing the complex CMOS neuronal units with emerging memristor-based neuronal devices as well as co-optimizing the parameters of sensors and EGTs should be carried out. Concepts such as in-sensor computing have been proposed and verified to be efficient in significantly increasing the integration density thus holding potential in constructing compact and powerful artificial perception systems.

Nevertheless, EGTs play essential roles in the future artificial intelligent perception area and are paving the way toward next-generation intelligent robotics, neural prosthetics, human-machine interaction, bioelectronics, and IoT technologies, etc. The realization needs continued and interdisciplinary collaborations among researchers with diverse backgrounds spanning from materials, biology, and computing engineering, etc.
